# Supplementary material for: Roles of protein–protein interactions and monolayer mechanics in tricellulin localization to tricellular tight junctions
Source: Biol Open. 2025 Sep 26;14(9):bio061987. doi: 10.1242/bio.061987 (PMC12505278; doi:10.1242/bio.061987)
Supplement: Supplementary information [file biolopen-14-061987-s1.pdf]

Supplementary
Figure 1

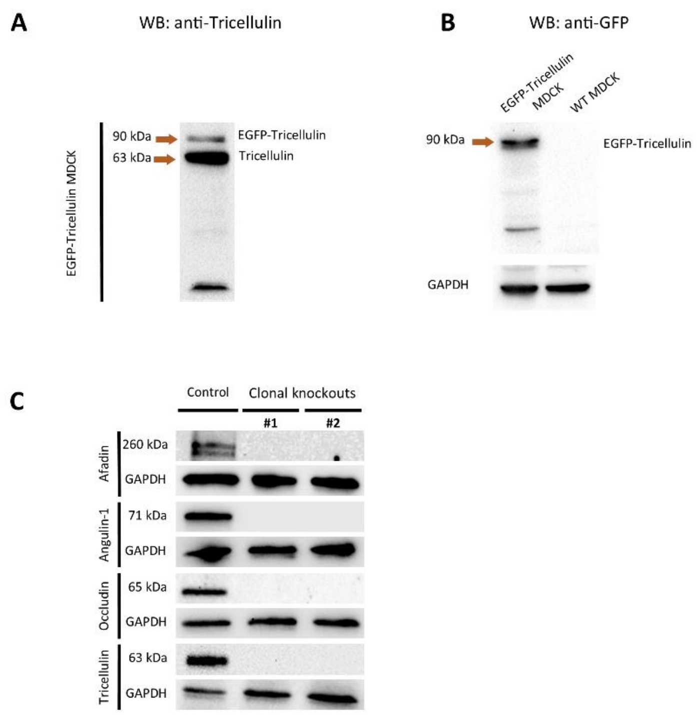

Fig. S1. Western blot analysis of a protein lysate from MDCK cell-line expressing EGFP-tricellulin probed with anti-tricellulin **(A)** and anti-GFP **(B)** antibodies. In panel 'B' also wild-type MDCK cell lysate was included as a negative control and GAPDH was used as a loading control. (C) Western blot analysis of afadin, angulin-1/LSR, occludin and tricellulin protein levels in lysates of control (wild-type MDCK cell) and corresponding two knockout MDCK cell clones. GAPDH was probed as loading control.

Biology Open • Supplementary information

Supplementary Figure 2

A **Angulin-1 Clonal Knockouts**

i

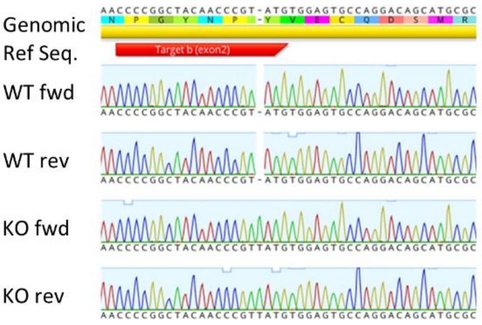

Angulin-1 KO Clone 1 Genomic DNA sequence

WT ..AACCCCGGCTACAACCCG-TATGTGGAGTGCCAGGACAGCATGCGC..
KO ..AACCCCGGCTACAACCCGTTATGTGGAGTGCCAGGACAGCATGCG...

Translated

WT ..NPGYNPYVECQDSMRTVRVVATKQGNAVTLGDYYQGRRITITGNADL..
KO ..NPGYNPLCGVPGQHAHGPGRGHQAGQCCDPGRLLPGPEDHHHRKC*

ii

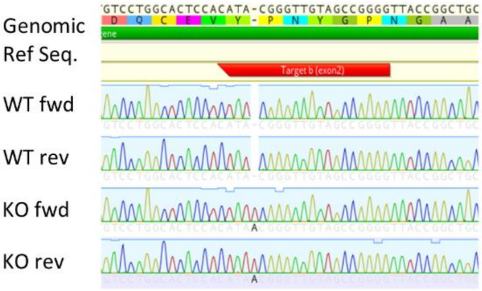

Angulin-1 KO Clone 2 Genomic DNA sequence

WT ..AACCCCGGCTACAACCCG-TATGTGGAGTGCCAGGACAGCATGCGC..
KO ..AACCCCGGCTACAACCCGATATGTGGAGTGCCAGGACAGCATGCG...

Translated

WT ..GYNPYVECQDSMRTVRVVATKQGNAVTLGDYYQGRRITITGNADL..
KO ..GYNPLCGVPGQHAHGPGRGHQAGQCCDPGRLLPGPEDHHHRKC*

B **Occludin Clonal Knockouts**

i

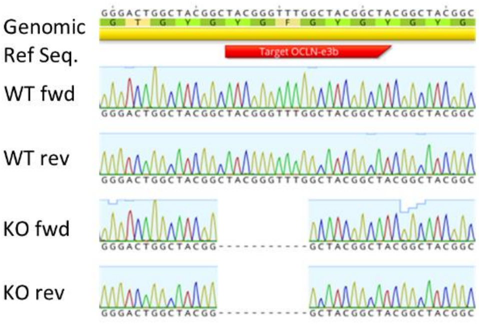

Occludin KO Clone 1 Genomic DNA sequence

WT ..GGGACTGGCTACGGCTACGGGTTTGGCTACGGCTACGGCTACGGC..
KO ..GGGACTGGCTACGG-----------GCTACGGCTACGGCTACGG...

Translated

WT ..GTGYGYGFGYGYGYGGYTDPRAAKGFLLAMVAFCFIAALVIFVTSVI..
KO ..GTGYGLRLRLRRLHGSQSSKGLPPGHGGLLFYRCIGDICYQRYKV*

ii

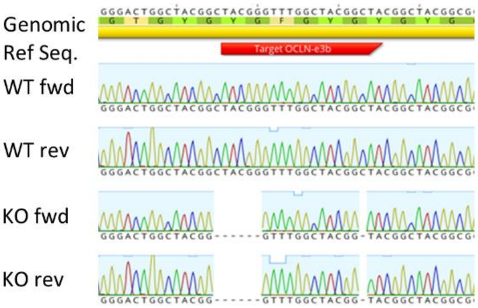

Occludin KO Clone 2 Genomic DNA sequence

WT ..GGGACTGGCTACGGCTACGGGTTTGGCTACGGCTACGGCTACGGC..
KO ..GGGACTGGCTACGG------GTTTGGCTACGG-TACGGCTACGGCG.

Translated

WT ..GTGYGYGFGYGYGYGGYTDPRAAKGFLLAMVAFCFIAA..
KO ..GTGYGFGYGTATAATRIPEQQRASSWPWWPFVLSLHW*

Fig. S2. (A) Sanger sequencing of genomic regions surrounding target sequences of the *angulin-1* gene (with forward and reverse primers) from wild-type (WT) and two angulin-1 KO clones (i, ii). sgRNAs were designed to target exon 2 of the *angulin-1* gene. Insertion of a single nucleotide A or T (highlighted red) resulted in a translational frameshift in exon 2, and a premature stop codon (indicated below in the amino acid sequence). **(B)** Occludin knockout clones, where exon 3 was targeted, contained (i) a deletion of eleven nucleotides (highlighted red) in one clone, and (ii) a six nucleotide and one deletion (highlighted in red). These resulted in premature stop codons (indicated below in the amino acid sequence).

Supplementary Figure 3

A **Tricellulin Clonal Knockouts**

i **Tricellulin KO** Clone 1 Genomic DNA sequence

Genomic DNA sequence

```
WT   ..ATCGTGTACGTCAACGACACCAA-CCGCGGGGGCCT..
KO1  ..ATCGTGTACGTCAACGACACCAACCCGCGGGGGCCT..   ——>  1 nt insertion   49,7%
KO2  ..ATCGTGTACGTCAACGACACCAA--CGCGGGGGCCT..   —-—>  1 nt deletion   49.8%
```

Translated

```
WT   ..IVYVNDTNRGGLCSYPLFNTPMNALLCRVEGGQVAALIFLFGITIVYLIGALVCLKLWRHHEVARRHRQYLEPREVS..
KO1  ..IVYVNDTNPRGPVLLPAVQHAHERAALPGRGGPGRRPHLPLRHHHRLPHRSLGLPEAVEARGGPEAQAVPGAAGGK*
KO2  ..IVYVNDTNAGACAPTRCSTRP*
```

ii **Tricellulin KO** Clone 2 Genomic DNA sequence

Genomic DNA sequence

```
WT   ..ATCGTGTACGTCAACGACACCAACC-GCGGGGGCCTGTGCTCCTACCCGCTGTTCAACACGCCC..
KO1  ..ATCGTGTACGTCAACGACACCA-----CGGGGGCCTGTGCTCCTACCCGCTGTTCAACACGCCC..   ——>  4 nt deletion   51.5%
KO2  ..ATCGTGTACGTCAACGACACCAACCCGCGGGGGCCTGTGCTCCTACCCGCTGTTCAACACGCCC..   —-—>  1 nt insertion   48.1%
```

Translated

```
WT   ..IVYVNDTNRGGLCSYPLFNTPMNALLCRVEGGQVAALIFLFGITIVYLIGALVCLKLWRHHEVARRHRQYLEPREVS..
KO1  ..IVYVNDTNPRGPVLLPAVQHAHERAALPGRGGPGRRPHLPLRHHHRLPHRSLGLPEAVEARGGPEAQAVPGAAGGK*
KO2  ..IVYVNDTNAGACAPTRCSTRP*
```

B **Afadin Clonal Knockouts**

i **Afadin KO** Clone 1 Genomic DNA sequence

Genomic DNA sequence

```
WT   ..CAGAGCAATGGACCCGAAAAGCAGGAGAAAGAAGGT...
KO1  ..CAGAGCAATGGACCCGAAA-----------GAAGGT..   ——>  11 nt deletion   51%
KO2  ..CAGAGCAATGGACCCGAAA-GCAG-AGAAAGAAGGT..   —-—>  2 nt deletion  49.8%
```

Translated

```
WT   ..QSNGPEKQEKEGVIQNFKRTLSKKEKKEKKKREKEAVRQASD..
KO1  ..QSNGPE---RRCYPELQENSLQERKEGEKEERKRGSEAGI**
KO2  ..QSNGPESRERRCYPELQENSLQERKEGEKEERKRGSEAGI**
```

ii **Afadin KO** Clone 2 Genomic DNA sequence

Genomic DNA sequence

```
WT   ..AAGGCTCAGAGCAATGGACCCGAAAAGCAGGAGAAAGAAGGTGTTATC..
KO1  ..AAGGCTCAGAGCAATGGACCCGAAA-GCAG-AGAAAGAAGGTGTTATC..   —-—>  3 nt substitution, and 2 bp deletion 58.1%
KO2  ..AAGGCTCAGAGCAATGGACCCGAAA-----------GAAGGTGTTATC..   ——>  11 nt deletion   41.9 %
```

Translated

```
WT   KAQSNGPEKQEKEGVIQNFKRTLSKKEKKEKKKREKEAVRQAS
KO1  KAQSNGPESRERRCYPELQENSLQERKEGEKEERKRGSEAGI*
KO2  KAQSNGPE---RRCYPELQENSLQERKEGEKEERKRGSEAGI*
```

Biology Open • Supplementary information

Fig. S3. (A) Next Generation Sequencing (NGS) of tricellulin knockout clones where exon 1 was targeted. Both analyzed clones contained two variants: (i) First clone contained one nucleotide insertion (49.7%, highlighted red) and one nucleotide deletion (49.8%, highlighted in red) variant. Total number of reads was 45 098. (ii) The second clone contained four nucleotide deletion (51.5%, highlighted red blanks) and one nucleotide insertion (48.1%, highlighted in red). Total number of reads was 181 209. All mutations led to premature stop codons (indicated below in the amino acid sequence). **(B)** Next Generation Sequencing (NGS) of afadin knockout clones where exon 3 was targeted. Both clones contained two variants: (i) Deletion of eleven nucleotides (51 %, highlighted in red) in one allele, and deletion of two nucleotides in the other allele (47.7%, highlighted in red). Total number of reads was 257 430. (ii) Substitution of three nucleotides and deletion of two nucleotides in one allele (58.1% highlighted red) and deletion of eleven nucleotides in the other allele (41.9% highlighted red blanks). Total number of reads was 257 430. All mutations result in a premature stop codon.

Supplementary
Figure 4

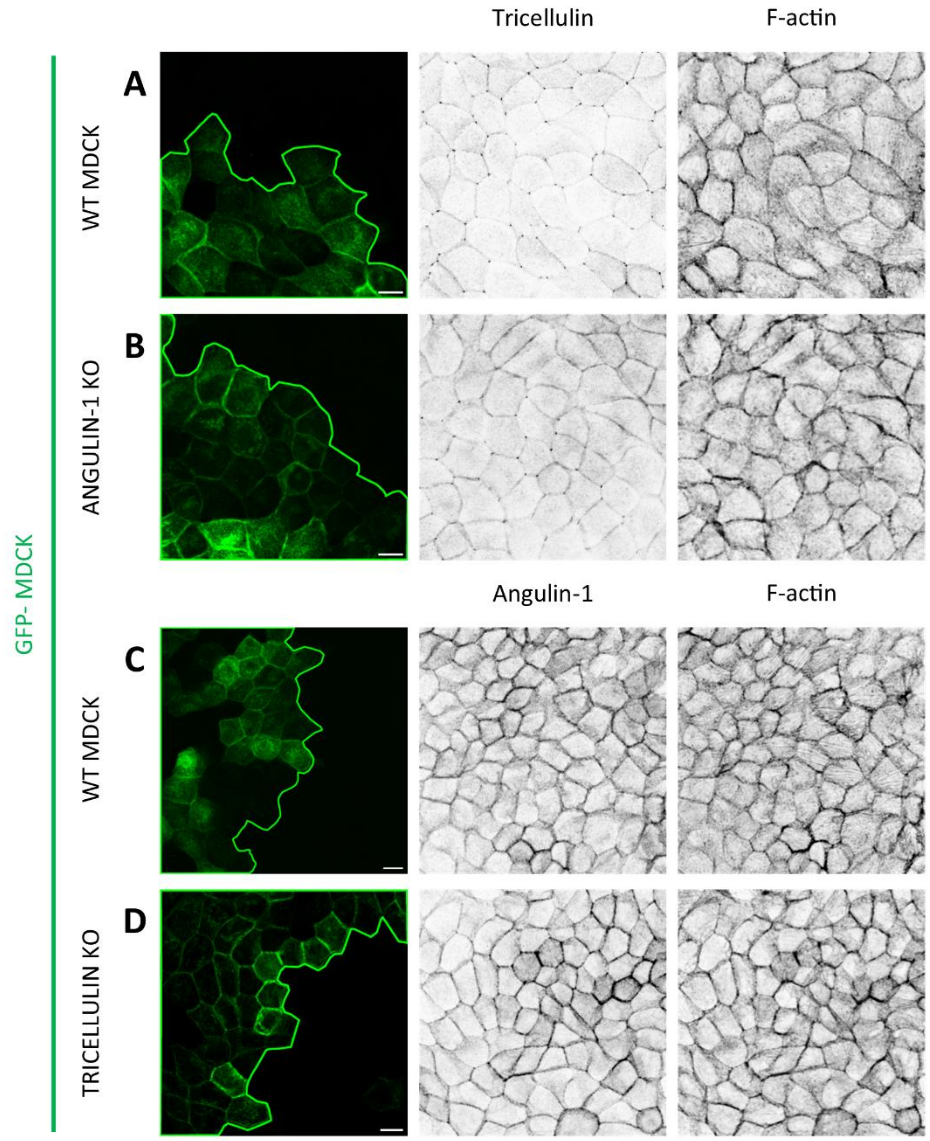

Fig. S4. Absence of angulin-1/LSR displaces tricellulin from the tricellular contacts, but loss of tricellulin does not affect the localization of angulin-1/LSR. Confocal microscopy images of GFP-expressing MDCK cells (green) co-cultured with WT MDCK cells (**A,C**), angulin-1 knockout cells (**B**), or tricellulin knockout cells (**D**). F-actin was detected by phalloidin staining, and tricellulin (**A-B**) and angulin-1/LSR (**C-D**) with specific antibodies. The green line in the images on left indicate the border between border GFP-expressing MDCK cells and 'non-fluorescent' wild-type or knockout MDCK cells. Scale bars, 10 μm.

Biology Open • Supplementary information

Supplementary
Figure 5

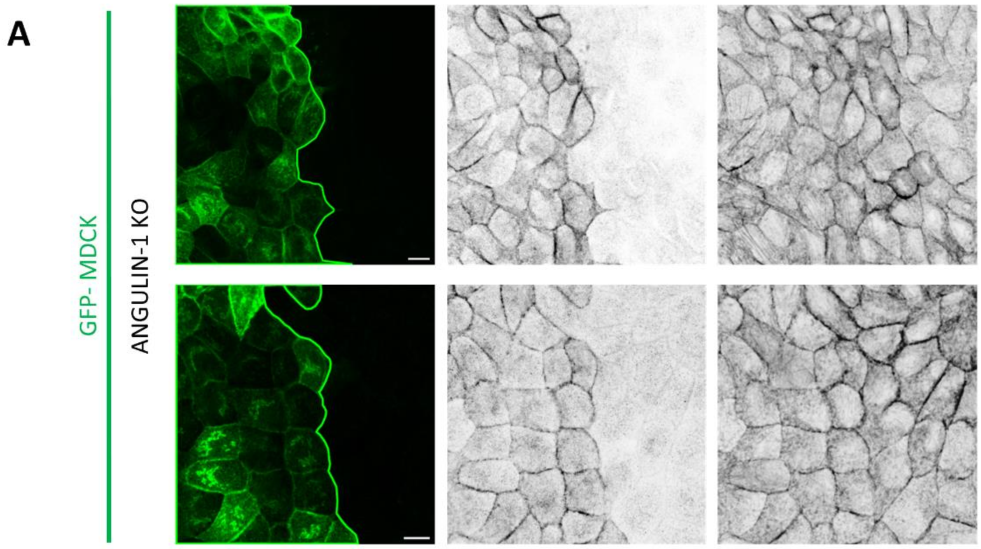

Fig. S5. Validation of the specificity of angulin-1/LSR antibody. Confocal microscopy images of GFP-expressing MDCK cells (green) co-cultured with angulin-1/LSR knockout cells. F-actin was detected by phalloidin staining (right), and the anti-angulin-1 antibody stainings are shown in the middle panels. Please note that anti-angulin-1 antibody stains cell-cell contact only in GFP-expressing wild-type cells, demonstrating the specificity of the antibody. Scale bars, 10 μm.

Supplementary
Figure 6

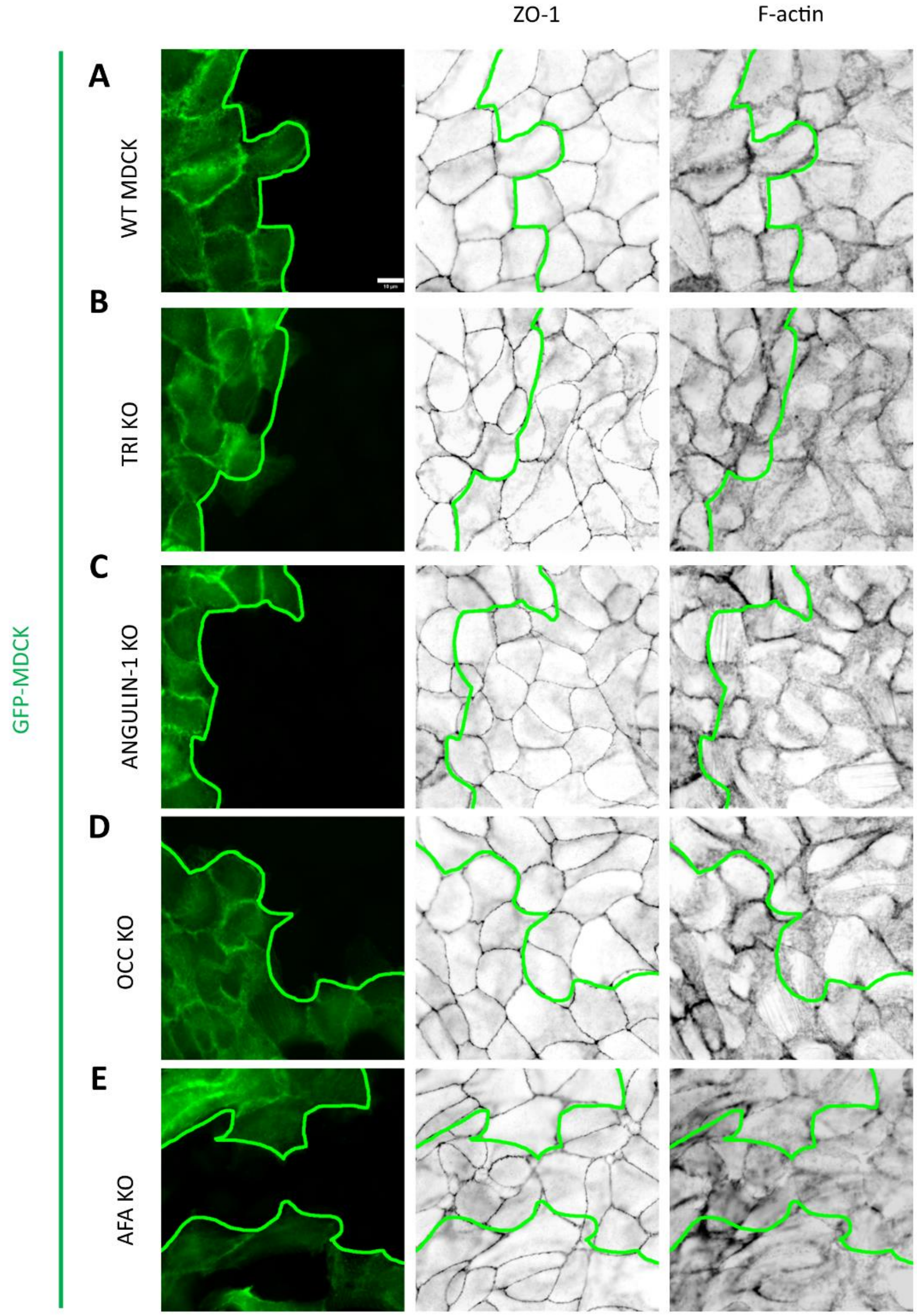

Fig. S6. Effects of tricellulin, angulin-1/LSR, occludin and afadin knockouts on tight Junctions (TJs). Wide-field microscopy images of GFP expressing MDCK cells (green) co-cultured with **(A)** wild-type MDCK cells, (B) tricellulin knockout, (C) angulin-1/LSR knockout, (D) occludin knockout, and (E) afadin knockout cells. Tight junctions were visualized by anti-ZO-1 antibody and F-actin with phalloidin staining. The green lines indicate the junctions between GFP-expressing wild-type MDCK cells and 'non-fluorescent' wild-type or knockout cells. Scale bars, 10 μm.

Table S1.

Available for download at
https://journals.biologists.com/bio/article-lookup/doi/10.1242/bio.061987#supplementary-data
